# “Conditional Restraints”: Restraining the Free Atoms in ARP/wARP

**DOI:** 10.1016/j.str.2008.12.011

**Published:** 2009-02-13

**Authors:** Wijnand T.M. Mooij, Serge X. Cohen, Krista Joosten, Garib N. Murshudov, Anastassis Perrakis

**Affiliations:** 1 Department of Biochemistry, The Netherlands Cancer Institute, Plesmanlaan 121, 1066 CX Amsterdam, The Netherlands; 2 Structural Biology Laboratory, Department of Chemistry, University of York, Heslington, York YO10 5YW, UK

**Keywords:** PROTEINS, CELLBIO

## Abstract

The automated building of a protein model into an electron density map remains a challenging problem. In the ARP/wARP approach, model building is facilitated by initially interpreting a density map with free atoms of unknown chemical identity; all structural information for such chemically unassigned atoms is discarded. Here, this is remedied by applying restraints between free atoms, and between free atoms and a partial protein model. These are based on geometric considerations of protein structure and tentative (conditional) assignments for the free atoms. Restraints are applied in the REFMAC5 refinement program and are generated on an ad hoc basis, allowing them to fluctuate from step to step. A large set of experimentally phased and molecular replacement structures showcases individual structures where automated building is improved drastically by the conditional restraints. The concept and implementation we present can also find application in restraining geometries, such as hydrogen bonds, in low-resolution refinement.

## Introduction

The determination of a protein structure by X-ray crystallography is a process that involves many different stages, from protein production, crystallization, and data collection, to structure solution and refinement. To increase throughput or to facilitate use by a wider (nonexpert) user base, automation remains an active field of research for all the steps involved. This is certainly the case for the final stages of structure determination: the building of a protein model. Over the years, much progress has been made in the field, exemplified in the development of software packages such as MAID (Levitt, 2001), Resolve (Terwilliger, 2003), Textal (Ioerger and Sacchettini, 2003), the implementation of the latter two packages in the PHENIX AutoBuild Wizard (Terwilliger et al., 2008), Buccaneer (Cowtan, 2006), ACMI (DiMaio et al., 2007), and ARP/wARP (Langer et al., 2008; Perrakis et al., 1999). For high-resolution datasets the task of constructing a protein model from an electron density map can now often be completed fully automatically. For low-resolution data, however, model building remains a largely manual and time-consuming procedure. So, there is a continuing need to extend the applicability of automated model-building methods to lower resolution.

The ARP/wARP software suite performs automated model building by coupling refinement with model building in an iterative fashion (Perrakis et al., 1999). In this approach, an electron density map is filled with “free” atoms. After initial model building, some of these atoms obtain a chemical identity. From then on, a hybrid model, consisting of free atoms and atoms that are part of a protein model, is iteratively refined and updated.

Free atoms serve two reasons in the ARP/wARP formulation: first, they are used to obtain better electron density maps through refinement; second, during model building they serve both as possible Cα guides for the protein backbone and as guides for the sequence assignment. In the case of high-resolution data, both these procedures work very well, as atoms will be recognizable in density, and enough observations for refinement are available. As a result, most of the free atoms are approximately in the right place. At lower resolutions, it becomes difficult to distinguish individual atoms. Also, it becomes difficult to refine the free atoms or hybrid model structure with the limited number of experimental observations and without chemical restraints. Thus, the errors in the positions of the free atoms become larger, and the electron density maps of significantly lesser quality. A way to remedy this situation is by adding structural information either in the context of the free atoms model or the hybrid model.

In addition to relying on the experimental information to guide the atoms to their desired position, the building and refinement process may be helped by pushing free atoms to positions that are likely, based on general knowledge of protein structure. Indeed, this is just what is done in standard restrained refinement, where a protein model is refined against the crystallographic data, under the geometric restraints of known bond length, angles, and torsion angles. Here, we want to extend this type of restraints to the free-atom part of the ARP/wARP model. Some very generic structural restraints can be added without sacrificing the ability of free atoms to “become” any protein atom. However, adding more specific restraints is at odds with the concept of chemically unassigned atoms. Conditional optimization provides one solution to this problem. In this approach, no final chemical assignments are ever made, but instead all possible assignments are considered simultaneously (Scheres and Gros, 2004). In the present work, which was inspired by conditional optimization, we implemented a less elaborate but computationally efficient approximation based on discrete model-building decisions. This is in line with the ARP/wARP framework, where a number of decisions on chemical assignments are made (e.g., in the main chain tracing as well as in the sequence docking stage). Despite these hard-decision steps, the overall process is tolerant to incorrect decisions: in the subsequent building step, ARP/wARP “forgets” its previous assignments, allowing for errors to be easily corrected.

This article describes the approach used in generating restraints for use in ARP/wARP, their implementation in REFMAC5 (Murshudov et al., 1997), and test results on the use of these restraints for datasets from the ARP/wARP Depot (Cohen et al., 2004).

## Results and Discussion

### Computational Methods

The ARP/wARP workflow has been described previously (Morris et al., 2003). In summary, it consists of a number of tasks that are performed in an iterative fashion, and briefly outlined as follows:

Start: The procedure typically starts by building a model of free atoms in an electron density map. In the case of a molecular replacement solution, alternative ways of starting are possible and are discussed elsewhere (Cohen et al., 2008).

Refinement: Refinement of the model is performed using the maximum-likelihood techniques as implemented in REFMAC5.

Model update: After refinement, the model is updated by adding and/or removing atoms. Atoms with a low electron density value in the 2mFo-DFc map or too far from all other atoms are removed; free atoms are added at positions of high density in the mFo-DFc map, provided they are close to existing atoms.

Model building: Pattern recognition techniques are employed to construct the protein model based on the current atomic coordinates and electron density maps. First, the main chain is built (Morris et al., 2002), and then efforts are made to identify the side chains using prior knowledge of the protein sequence (sequence docking).

In the classic ARP/wARP system, the number and sequence of these steps was fixed (Perrakis et al., 1999). Recently a new, more adaptive control system was developed (Cohen et al., 2004), where the order and number of steps is dependent on the progress of the refinement and model building.

In the current work, the task of generating restraints that are specific for free atoms is added to the workflow. This task is performed in each refinement step (with the exception of the initial refinement before the first model building). The restraints generated are subsequently used in a REFMAC5 refinement through a newly implemented functionality that allows the specification of restraints on a per-atom basis. Here, we first describe the restraint functions that were implemented in REFMAC5 to facilitate conditional restraints. We then describe in detail how conditional restraints are generated for two cases: one applicable to all free atoms and one applicable at the interface between the protein atoms and the free atoms in the hybrid model.

#### Implementation of External Restraints in REFMAC5

To facilitate the application of conditional restraints we implemented the following functions in the macromolecular refinement software REFMAC5 (Murshudov et al., 1997). All of these restraints can be specified with a simple syntax in a text file that can be read by REFMAC5.

**Figure 1:**
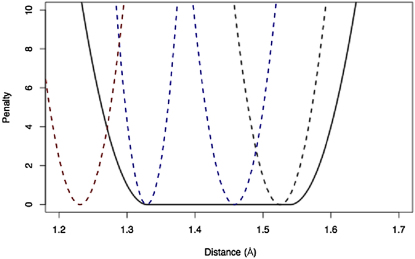
Graphical Comparison of the Functions Used for Quadratic and Interval Restraints The function used for the interval restraint is shown with the default values as a solid black line. The quadratic restraints for all main chain bonds are shown as dashed lines; black in the Ca-C, blue the N-Ca and C-N bonds, and red the C = O bond.

Quadratic restraints have the general form

(1) $$\frac{1}{\sigma_{q}^{2}}{\left(q_{m}-q_{i}\right)}^{2}$$

where *m* stands for model and *i* for ideal; *q* stands for the restrained quantity (distance, angle, or chiral volume) and σ for the associated expected standard deviation. These restraints are typically used in macromolecular refinement to restrain quantities such as bonds and angles to known values ( Figure 1).

Interval restraints have the general form

(2) $$\{\begin{array}{ccc} \frac{1}{\sigma_{il}^{2}}{\left(q_{m}-q_{l}\right)}^{2} & \text{if}q_{m}<q_{l} & \\ 0 & \text{if}q_{l}<q_{m}<q_{h} & \\ \frac{1}{\sigma_{ih}^{2}}{\left(q_{m}-q_{h}\right)}^{2} & \text{if}q_{m}>q_{h} & \end{array}$$

and were implemented for bonds and distances. In interval restraints instead of an ideal value for each quantity, if the measured value (*q_m_*) becomes less than a lower bound of the defined interval (*q_l_*) then restraints with strength *σ_il_* are applied; if *q_m_* is higher than *q_h_* then a restraint with strength *σ_ih_* is applied; if the distance is within the interval (*q_l_* … *q_h_*) then no restraint is applied. Note that this function is continuous up to the first derivative with respect to *q_m_*, whereas the second derivative is discontinuous. Interval restraints have the advantage of giving equal preference to a number of values, while disallowing others (Figure 1). They are well suited for restraining (e.g., chemical bonds) when it is not known exactly what the atoms are and thus the ideal expected value for that chemical bond.

Planarity restraints have the general form

(3) $$\frac{1}{\sigma_{p}^{2}}\sum_{j=1}^{n}{\left(ax_{j}+by_{j}+cz_{j}+d\right)}^{2}$$

where are *a*, *b*, *c*, and *d* are coefficients of the plane that is least-squares fit to the *n* atoms that must be on the same plane. Because these coefficients are calculated using the same atoms, they are functions of the atomic position, and in derivative calculation this is taken into account.

Repulsion restraints have the form

(4) $$\{\begin{array}{cc} \frac{1}{\sigma_{v}^{2}}{\left(d_{m}-d_{i}\right)}^{2} & \text{if}d_{m}<d_{i}\\ 0 & \text{otherwise} \end{array}$$

where the ideal distance, *d_i_*, is atom pair specific and depends on atom type and the nature of interaction between them. If both atoms are free atoms, then *d_i_ = 2r_free_* (where *r_free_* is the default van der Waals radius for free atoms and is equal to 0.6). If one of the atoms is free and another is from the macromolecular model then the distance for activation of repulsion restraint is equal to *max*(*d_inc_, r_prot_+r_free_-d_inc_*), where *r_prot_* and *r_free_* are the van de Waals radii protein atoms and free atoms, respectively, and *d_inc_* is a default minimal value (0.7 Å). Similar to interval restraint, this function is continuous up to the first derivative, and the second derivative is discontinuous. If two atoms are bonded with covalent (or free-atom) bonds, then there are no repulsion restraints between them. Similarly, atoms that are two bonds apart do not contribute to repulsion restraints. If atoms are three bonds are apart (torsion angle-related atoms), then their van de Waals radius is reduced (by 0.3 Å).

#### General Restraints between Free Atoms

##### Bond Distance Restraints

General distance restraints can be applied to all free atoms. Protein geometry dictates that no atom should have more than three bonded neighbors, and all bonded atoms should be in the range 1.2–1.6 Å. At the same time, the closest nonbonded atoms cannot be closer than 2.5 Å from each other. If two atoms lie between 1.6 Å and 2.5 Å from each other, this is most likely wrong—the only exceptions in proteins are sulphurs in disulphide bonds and metal ions. If during the bond restraint assignment more than three neighbors are found within bonding radius, the closest neighbors are chosen and assigned as bonded. An additional requirement in the selection of bonds is that three-membered or four-membered rings are not allowed. If such a cyclic structure would be generated, the longest bond in this ring is removed. Free atoms that are bonded are given an interval distance restraint. The allowed region for the interatomic distance is set to 1.34–1.53 Å, which spans all main chain bond length with the exception of the C = O bond and the vast majority of other bonds in a protein structure. Outside this region a restraint with a standard deviation of 0.03 Å is applied.

##### Bond Angle Restraints

Any atom in a protein is either sp^2^ or sp^3^ hybridized, dictating that bond angles should be either ∼109.5° or ∼120°. This information is exploited in the context of free atoms by interval distance restraints between free atoms that are separated by two bonds. The allowed range of distances is set to 2.34–2.65 Å (these bounds are based on the distance tables by Scheres and Gros [2003]). Outside this range, distance restraints with a standard deviation of 0.06 Å are applied.

##### Repulsion Restraints

Free atoms with three bonds should not get any more neighbors, and to this end such atoms are given an increased van der Waals radius (1.2 Å instead of the usual 0.6 Å for free atoms, which allows them to make bonds without repulsion), to push any further neighbors away.

##### Planarity Restraints

Because proteins do not contain rings of sp^3^ atoms (with the exception of proline), it is reasonable to assume that any small cyclic structure formed within the free-atom model should be planar. To this end, all five- and six-membered rings in the free-atom part of the model are identified, and a planarity restraint is added for the atoms in such rings. In addition, one to three neighbors in the rings are assigned appropriate bond angle restraints, depending on the type of the ring (108° or 120°, σ = 3.0°), instead of the previously mentioned interval restraints. Rings of seven, eight, or nine atoms are broken by removing the bond distance restraints for the longest bond in such rings.

#### Specific Restraints between Protein and Free Atoms

For the more-specific restraints at the interface between the protein and the free-atom part of ARP/wARP's hybrid model, four points of extension of the protein model are considered. Restraints to the dummy atoms are then based on a putative chemical assignment of the free atoms that are close by.

##### Cα's Carbon Atoms with Unknown Residue Type

The initial step of the model building in ARP/wARP is to construct a main chain trace. When subsequent sequence docking for a certain main-chain segment fails, this segment will remain in the model as a polyglycine peptide. For each residue in these undocked parts of the protein model, the dummy atoms within 1.5 Å of the calculated Cβ position are identified. For the atom closest to the ideal Cβ position, bond distance and angle restraints are generated based on normal protein (Engh and Huber, 1991) geometry targets. Also, a chiral volume restraint for the Cα atom is added.

##### Amide Nitrogen Atoms of Undocked Chains

At this position a free atom may be bonded if the residue is a proline. To this end, if a free atom closer to the ideal Cδ position exists (within 1.5 Å), it is checked for whether it is involved in a five-member ring involving the amide N and Cα. If so, bond and angle restraints are added for this dummy (Cδ) atom, and a planarity restraint for the amide N atom.

##### C Termini of Main-Chain Segments

At the endpoints of all main-chain segments (either in sequence or undocked), the position of the next amide N atom is calculated, and the free atom closest to it (within 1.5 Å) is identified. Normal protein bond distances and angles are assigned to that free atom. Next, the putative next Cα atom is calculated, and free atoms closest to it (within 1.5 Å) are identified, assuming a *trans* peptide bond. For this atom again, bond length and angle deviations are added, as well as a torsion restraint for the putative amide bond (180°, σ = 5°).

##### N Termini of Main-Chain Segments

An ideal position for the preceding carbonyl C atom cannot be calculated. Instead, the atom closest to any position at the correct bond length and angle (but with unspecified torsion—i.e., a circle on a cone) is identified. This closest atom (within 1.5 Å) is then assigned bond length and angle restraints appropriate for a backbone carbonyl carbon atom. Then two bonded free atoms are searched for, that could be Cα and O atoms (within 1.5 Å of their ideal positions), assuming a *trans* peptide bond. Again, Engh-Huber restraints are applied to their bonds and angles involving the putative C atom and the protein atoms (Engh and Huber, 1991). Additionally, a torsion restraint for the new peptide plane is added, and any additional bonds on the putative O atom (other than to the putative C) are removed.

#### On the Choice of Parameters for Conditional restraints

The ideal (or target) values for all restraints were based on chemical knowledge about proteins. Most values used in this study were extracted from the REFMAC5 dictionary. The expected standard deviations were optimized by trial and error in a small “training set” of structures (two to five at different stages of the project), which was not part of the test set. These values are tabulated in Table S1, available online. We chose to implement all restraints that made chemical sense for atoms of unknown identity; thus, no detailed study of the effect of the different categories of restraints was made. At the same time, we could not find any category of restraints that would be systematically violated and therefore we did not eliminate any of our original ideas in the course of development and testing. It should be noted that our restraints are valid only for protein structures. In the presence of nucleic acids, for example, assuming planarity of six-membered rings would clearly be incorrect. One should also consider treating separately the double-ring systems of pyrimidines, and possibly the purine rings as well. Such considerations might be facilitated by recent developments in the ARP/wARP software (Hattne and Lamzin, 2008).

#### Free-Atom Removal

In addition to generating restraints for REFMAC5 refinement, the restraints program “weeds out” some free atoms around the protein part of the model. This is only performed after a new cycle of main-chain building and sequence docking. In further atom update and refinement steps we allow the building of new free atoms close to any protein atom, to facilitate the correction of any wrong assignments made in the main-chain/side-chain building step.

The atom removal is performed in various layers. First, all atoms closer than 1.0 Å to any protein atom are removed, unless they are selected to be directly bonded to the protein (i.e., to any of the protein extension points described previously). Then, atoms closer than 2.0 Å are removed unless they are separated from a protein atom with less than three bonds. Last, atoms closer than 2.5 Å to any protein atom are removed unless they are separated from a protein atom by less than four bonds.

#### Composition of the Test Set

The effect of restraints on the automated model building was tested on a number of datasets from the ARP/wARP Depot (http://xtal.nki.nkl/Depot). Depot holds various user-contributed cases for both experimentally phased structures and molecular replacement solutions, with the initially obtained maps and the final models. The Depot contains a few structures from Structural Genomics initiatives, without being dominated by them, because these tend to be much better data than the average for that resolution. The test set contains structures over a wide spectrum of resolution, with less emphasis on high-resolution structures, and is thus designed to challenge rather than to flatter ARP/wARP performance. All available experimentally phased structures from Depot that were not complexes were used. From the molecular replacement cases, a subset of structures was used where the sequence identity and/or the completeness of the MR search model was less than 70% of the final model. This selection was done to prevent testing the method on essentially correct molecular replacement solutions. The use of restraints was added to the *warp_tracing.sh* shell script of ARP/wARP release 7.1-beta. This script runs 10 cycles of model (re)building, with five cycles of atom update and REFMAC5 refinement in between. Restraints for the free atoms were generated before each call to REFMAC5, with the exception of the first call prior to the initial model building. For comparison, otherwise-identical runs were performed with the use of conditional restraints switched off. Results were analyzed comparing the number of Cαs in the automatically build model to that in the known final structure. We refer as “correct Cα” to a Cα that lies within 1 Å of a Cα in the final model and has correct peptide directionality. We also compare the number of the Cαs with correctly identified side chains and refer to them as “correct residues.”

#### Implementation and Availability

The code to generate a restraints dictionary for input in REFMAC was written in Java and makes use of the OpenAstexViewer library (Hartshorn, 2002; http://openastexviewer.net/). The use of atom-specific restraints in protein structure refinement was implemented in REFMAC, and this functionality is available now in version 5.5. Here we used version 5.5.0054. The restraints methods will be available from release 7.1 of the ARP/wARP package in 2009.

### Overall Performance

**Figure 2:**
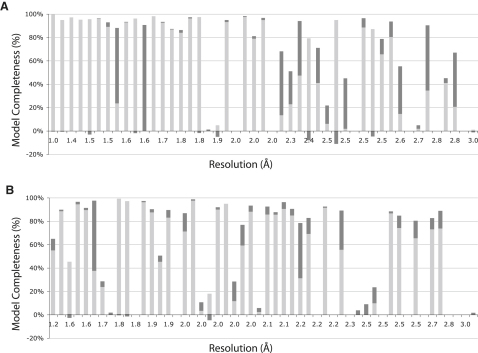
Analysis of the Performance of ARP/wARP Model Building with and without Conditional Restraints Test sets are ordered by resolution. The light gray bar indicates the percentage of correct residues as defined in text. The improvement using conditional restraints is depicted as a darker gray bar; in the cases that conditional restraints led to a worse model, the difference is noted as a negative bar. (A) Results for the experimentally phased cases. (B) Results for the molecular replacement cases.

The test set contains 47 experimentally phased and 52 molecular replacement structures. For the 47 experimentally phased structures, the average performance of ARP/wARP with conditional restraints increases from 67% to 74% correct Cαs, and from 54% to 66% correct residues (summarized in Figure 2A). For the 52 molecular replacement test cases, the number of correct Cαs increases from 54% to 59%, and of correct residues from 47% to 54% (summarized in Figure 2B). One could thus conclude that the overall effect of conditional restraints was a medium improvement in the completeness of the final models. These averages, however, do not reflect the large effects observed for individual cases.

### Conditional Restraints Starting from Experimental Maps

**Figure 3:**
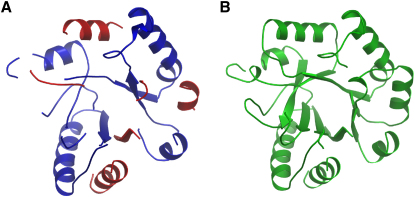
Differences in Model Completeness Using Classic ARP/wARP and Conditional Restraints (A) Red areas indicate the main chain is not docked in sequence. (B) All the main chain found was docked in sequence. Pictures made with OpenAstexViewer.

For the experimentally phased structures test cases at higher resolution (<2.0 Å), conditional restraints show the most dramatic improvement for a small protein at 1.6 Å with an average initial phase error of 60°. Without the use of conditional restraints, ARP/wARP fails to improve on the initial electron density map; the automated building does not progress from the initial model. With conditional restraints, however, the complete protein is automatically built. Additional improvements are in the low-resolution 2.2–2.8 Å range, with two cases that conditional restraints lift from the 40%–60% completeness region (which is a model hardly useful for the end user in practice) to models more than 90% complete. In several other cases the model completeness increases between 10% and 50%, resulting in significantly more complete models in low resolution. A noteworthy example is observed for a protein at 2.4 Å, where the use of conditional restraints results in a nearly complete model (more than 90% of the Cαs built and residues assigned), where without conditional restraints, only three-quarters of the Cαs is built and only half of the residues was correctly identified. This difference in performance is depicted in Figure 3. As can be seen, the use of conditional restraints leads to a number of β strands in the core of the protein being built, additional loops being built, and various strands and helices being extended, compared with the ARP/wARP run without conditional restraints. Note that the use of conditional restraints does not have adverse effects in model completeness, and this result justifies their use as default in ARP/wARP.

### Conditional Restraints Starting from Molecular Replacement

For the molecular replacement cases the biggest improvement is observed for a structure at 1.65 Å. Here, the molecular replacement search model was around half of the protein under consideration. The initial model building step in ARP/wARP finds ∼30% of Cαs and residues. Without restraints, after 10 cycles this increases to ∼60% and 40% respectively. With the use of conditional restraints, an essentially complete model is built. Drastic results are also observed for a case at 2.3 Å where initial model building finds 60% of the Cαs and 45% of residues. Without restraints, not much is added to this (65% and 55%); with restraints, the final model is almost complete, encompassing 90% correct Cαs and residues. Other cases show modest but significant improvements in model completeness. Again, adverse effects of the use of restraints hardly occur.

### Conditional Restraints Performance as Function of Resolution and Phase Error

**Figure 4:**
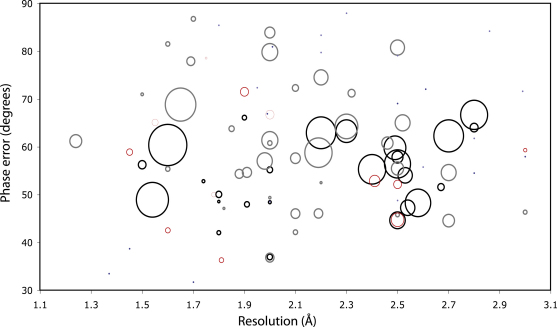
Analysis of the Improvements Achieved by the Use of Restraints as a Function of Resolution and Phase Error Black and gray circles denote an increase in the percentage of correct residues for the experimentally phased and the molecular replacement test cases respectively; red and washed-out red circles denote a decrease. In both cases, the observed change is proportional to the area of the circle. Blue and washed out blue dots denote no change.

In Figure 4 we display the improvements achieved by the use of conditional restraints plotted against resolution and the phase error of the starting map (which is known because we have the final model from which we can calculate final phases). For the experimentally phased test set, the majority of cases where conditional restraints are beneficial are in resolution between 2.2 and 2.8 Å, whereas for the molecular replacement set, there is no clear resolution range. In terms of phase error, however, it is now clear that all cases fall roughly within a range of 45° to 70° phase error. This trend can be explained. If the initial model has low phase error, the restraints have no effect, and for these cases the model can already be built completely by ARP/wARP without them. On the other extreme, for very large phase errors, ARP/wARP can hardly build any residues, and hence the use of restraints does not rescue anything because the information added is not sufficient to improve the phases to such an extent that it leads to an interpretable model. However, at the borderline of density that is still (in varying degrees) interpretable by ARP/wARP, conditional restraints have a large positive effect in the completeness of the models build by ARP/wARP.

### Application of Conditional Restraints

The addition of conditional restraints for ARP/wARP's free atoms can significantly help the automatic interpretation of electron density maps by the automated model building and refinement procedure. The present study indicates there is little if any reason not to use conditional restraints on free atoms routinely in ARP/wARP. Given an individual case, it may do nothing, but it may also be the difference between building the structure automatically or not.

We are currently working on various ideas to increase the impact of the use of these restraints. One of the approaches aims to harvest information available from the sequence docking. When sequence docking fails, it may be possible to still obtain reasonable guesses for at least a number of individual side chains. Placing new free atoms according to such side-chain assignments may improve the generated restraints because the bonding environment for such newly placed atoms will then consist of chemically sensibly geometries.

Conditional restraints, and in particular the interval distance restraints, pave the way for two more general applications. First, the conditional interval restraints described previously, unlike quadratic penalty restraints, are well suited for hydrogen bonding distances, which are especially important in low-resolution refinement. One needs to add to the current conditional restraints module a functionality that in each cycle of refinement could derive upper and lower bounds for potential hydrogen bonds and feed them to the refinement module. Furthermore, discrete (conditional) decisions after each refinement step would be important to allow for conformational changes during refinement and new bonds formed and others to be broken. Second, the same technique can be applied for transferring information from a higher-resolution structure to a lower-resolution one. That may dramatically improve behavior of refinement at low resolution and help to extract biologically significant information from limited and noisy data. One can foresee potential application of this to X-ray structure analysis of a macromolecular complex or multidomain proteins for which high-resolution structures of component proteins or domains are available. Again, the conditional idea whereby the applicability of specific restraints is updated in each step, combined with interval penalty restraints to allow restraining long-distance features of the high-resolution structure to the lower resolution one, is likely to be of benefit.

## Supplemental Data

Document S1. One Table
