# Supplementary material for: “Conditional Restraints”: Restraining the Free Atoms in ARP/wARP
Source: Structure. 2009 Feb 13;17(2-3):183–9. doi: 10.1016/j.str.2008.12.011 (PMC2670983; doi:10.1016/j.str.2008.12.011)
Supplement: Document S1. One Table [file mmc1.pdf]

Supplemental Data

"Conditional Restraints":

Restraining the Free Atoms in ARP/wARP

Wijnand T. M. Mooij, Serge X. Cohen, Krista Joosten, Garib N. Murshudov, and Anastassis Perrakis

Table S1. The Paramters for All Restraints Used in the Implementation of "Conditional Restraints"

	Type	Ideal value(s)	σ
General			
Bonds	Interval	1.34 - 1.53 Å	0.03 Å
Angles	Interval	2.34 - 2.65 Å	0.06 Å
Rings	Planarity	planar	0.05 Å
Angles 5-membered	Quadratic	108.0^o	3.0^o
Angles 6-membered	Quadratic	120.0^o	3.0^o
4>-membered<br>7=<-membered	Broken		
Repulsion	Repulsion	1.2	
Protein-free			
$C\alpha_i$			
Ca_i-$C\beta_{dum}$	Quadratic	1.53	0.02
N_i-Ca_i-$C\beta_{dum}$	Quadratic	110	2
C_i-Ca_i-$C\beta_{dum}$	Quadratic	110	2
Ca_i-N_i-C_i-$C\beta_{dum}$	Chirality	2.5	0.1
N_i (if likely Pro)			
N_i-$C\delta_{dum}$	Quadratic	1.47	0.02
$C\alpha_i$-N_i-$C\delta_{dum}$	Quadratic	112.0	2.0
C_{i-1}-N_i-$C\delta_{dum}$	Quadratic	125.4	3.0
C_{i-1}-N_i- $C\alpha_i$-$C\delta_{dum}$	Quadratic	planar	0.05
C_{ter}			
C_{ter}-N_{dum}	Quadratic	1.33	0.02
N_{dum}-$C\alpha_{dum}$	Quadratic	1.46	0.02
$C\alpha_{ter}$-C_{ter}-N_{dum}	Quadratic	116.5	2.0
O_{ter}-C_{ter}-N_{dum}	Quadratic	123.0	2.0
C_{ter}-N_{dum}-$C\alpha_{dum}$	Quadratic	121.7	2.0
$C\alpha_{ter}$-C_{ter}-O_{ter}-N_{dum}-$C\alpha_{dum}$	Planarity	planar	0.05
N_{ter}			
N_{ter}-C_{dum}	Quadratic	1.33	0.02
C_{dum}-O_{dum}	Quadratic	1.23	0.02
C_{dum}-$C\alpha_{dum}$	Quadratic	1.52	0.02
$C\alpha_{ter}$-N_{ter}-C_{dum}	Quadratic	121.7	2.0
N_{ter}-C_{dum}-O_{dum}	Quadratic	123.0	2.0
N_{ter}-C_{dum}-O_{dum}	Quadratic	116.2	2.0
O_{dum}-C_{dum}-$C\alpha_{dum}$	Quadratic	110.0	2.0
$C\alpha_{ter}$-N_{ter}-C_{dum}-O_{dum}-$C\alpha_{dum}$	Planarity	planar	0.05

σ stands for the associated expected standard deviation of ideal values. The formulas of each type of restrain are in the text; the number in parenthesis refers to the number of the formula in the text.
